# Thermal gradient induced tweezers for the manipulation of particles and cells

**DOI:** 10.1038/srep35814

**Published:** 2016-11-17

**Authors:** Jiajie Chen, Hengji Cong, Jacky Loo, Zhiwen Kang, Minghui Tang, Haixi Zhang, Shu-Yuen Wu, Siu-Kai Kong, Ho-Pui Ho

**Affiliations:** 1 Department of Electronic Engineering, The Chinese University of Hong Kong, Shatin, N.T., Hong Kong SAR, China; 2 Biochemistry Programme, School of Life Sciences, The Chinese University of Hong Kong, Shatin, N.T., Hong Kong SAR, China

## Abstract

Optical tweezers are a well-established tool for manipulating small objects. However, their integration with microfluidic devices often requires an objective lens. More importantly, trapping of non-transparent or optically sensitive targets is particularly challenging for optical tweezers. Here, for the first time, we present a photon-free trapping technique based on electro-thermally induced forces. We demonstrate that thermal-gradient-induced thermophoresis and thermal convection can lead to trapping of polystyrene spheres and live cells. While the subject of thermophoresis, particularly in the micro- and nano-scale, still remains to be fully explored, our experimental results have provided a reasonable explanation for the trapping effect. The so-called thermal tweezers, which can be readily fabricated by femtosecond laser writing, operate with low input power density and are highly versatile in terms of device configuration, thus rendering high potential for integration with microfluidic devices as well as lab-on-a-chip systems.

Trapping and manipulation of micrometer-sized dielectric particles by single-beam optical gradient forces^1^ have benefited many applications in physics and biochemistry^2^. However, typical optical tweezers often require cumbersome optical setups, high numerical aperture lens and high laser intensity. Recently, the study of optical tweezers based on plasmonic nanostructures has led to a remarkable improvement. Plasmonic trapping devices such as silver nanostructure on tapered fiber^3^ or periodic gold discs^4^ have shown the low laser threshold intensity and localisation accuracy beyond the diffraction limit. However, plasmonic thermal heating due to electrical resistance in metals has been a major concern of plasmonic trapping^5^, and various techniques including self-induced back-action (SIBA) trapping^6^,^7^, integration of heat sinks^8^ and drastic reduction of sample chamber thickness^9^,^10^ have been used to alleviate such thermal effects. In spite of this, it is also exciting to see that optical thermal effect can be utilized to facilitate trapping. Plasmonic bowtie nano-antenna arrays^11^, single plasmonic nano-antenna assisted with an AC electric field^12^, thermal absorption medium^13^, random plasmonic absorption structures^14^,^15^, and continuous gold films^16^, have been reported to perform manipulation and transportation of small colloid particles through the assistance of plasmon-induced thermal convection and diffusion. In addition, fibre-optic based tweezer systems^17^,^18^,^19^,^20^,^21^, which are readily integrated into lab-on-a-chip devices, can also reduce the exposure intensities and eliminate the necessity of bulky optics. However, most existing devices still require optics system to achieve focusing and alignment with sufficiently high precision or an AC field to assist the trapping, which are not favourable for integration with microfluidic devices and most biomedical applications^22^,^23^,^24^,^25^. In addition, despite that the optically induced thermal tweezers have been studied previously^26^,^27^, the true origin of thermally induced local forces has not been fully explored.

Here, without the use of cumbersome optics or complex optical field enhancement structures, we demonstrate photon-free trapping of dielectric polystyrene spheres (PSs) as well as live *E. coli*, which has a refractive index (1.38) comparable to water, using a micro-scale electric thermal heater (METH). The METH device is fabricated from a continuous gold thin film by means of direct writing with a femtosecond laser. A thin SiO_2_ overlay coating is applied to the surface to ensure electrical isolation from the aqueous sample to be placed on the top. The operation of trapping with METH is entirely due to thermal effects. When the METH device is energised with a DC current, a temperature profile is created around the device. It turns out that thermal convection and thermophoresis induced by temperature gradient around the METH can lead to a net trapping force that keeps the target particles inside a small volume. The convection generated from METH can be classified as single cell Rayleigh-Bénard convection^28^, which originates from the movement of rising fluid from the heated METH device surface, with continuity maintained by radial in-flow from the surrounding. The flow entering and leaving the hot zone is typically in a toroidal shape. When the size of the METH device is in micrometer scale, it can generate convective flow similar to that induced by optical absorption^16^ or thermo-plasmonic absorption, which occurs with velocities in the order of micrometer per second^29^,^30^. When a DC electric current is applied to the device, convective flow brings target particles into the hot zone along a radially inward direction. A second force component, primarily associated with thermophoresis due to the temperature gradient, and with a correct sign, may be sufficiently large to counteract the convective drag force. Consequently, a stable trap is established in the hot zone directly above the METH device. Because of thermophoresis, the colloidal particles first attain a drift velocity of 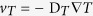, where D_T_ is the thermal diffusion coefficient, which also leads to a particle concentration gradient 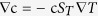, where S_T_ = D_T_/D is called the Soret coefficient and D is the Brownian diffusion coefficient. Thermophoresis is an interfacial phenomenon caused by thermal gradient induced particle/solvent interface inhomogeneity^31^. While the measurement and origin of thermophoresis are currently attracting much research interest^32^,^33^,^34^, it is commonly believed that the Soret coefficient is susceptible to the influence of particle size^35^, temperature^36^, ion concentration and pH value^37^. In our trapping scheme, we have a negative S_T_^35^,^38^,^39^, so that particles are trapped in the hottest region directly above the METH device. In addition, METHs with longer sizes also exhibit good trapping performance. We have further investigated the influence of particle size and ion concentration in order to obtain a quantitative estimation of the thermophoresis effect in our scheme. Through simulation studies, we obtain the temperature distribution and temperature gradient around the device. Our results show that typical power density of our METH tweezers is in the range of 10−100 μW/μm^2^, which is lower than the optical power density of plasmonic assisted trapping (≥100 μW/μm^2^)^11^. We expect a relatively lower chance of sample damage^9^,^14^,^15^,^16^,^22^ when trapping is performed with METH. This can be explained by the fact that plasmonic optical trapping results in temperature increase in the trapped object due to (i) radiation absorption by the object itself and (ii) conductive thermal energy from plasmonic absorption in the gold nanostructure. Whereas in the case of METH, trapping is entirely driven by localised resistive heating, which means that the trapped object only needs to endure one type of heating, and it is of relatively lower threshold level. With it being “photon-free”, the reported thermal tweezers will not be affected by refractive index or absorption in the target. Moreover, one can readily fabricate a large number of METH devices in 2-dimensional arrays for performing trapping and manipulation of a number of target objects.

## Materials and Methods

### Device fabrication

**Figure 1:**
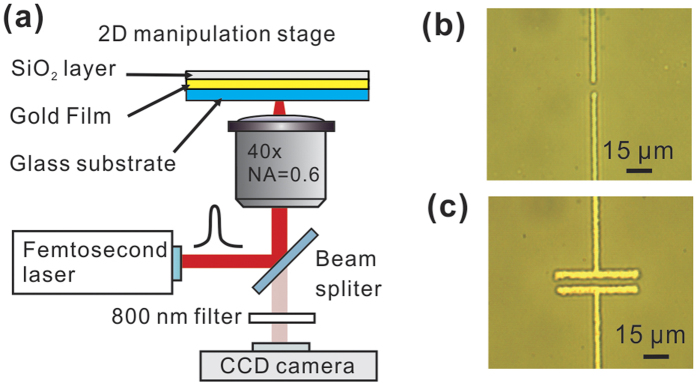
(**a**) Setup of using a femtosecond laser to perform METH fabrication. Βright field microscopy of fabricated device: (**b**) Point-like METH; (**c**) 50 μm long METH.

To fabricate the structure, we first coated a continuous gold thin film on microscope cover glass (22 × 22 mm, 0.13–0.16 mm thick, Ted Pella Inc.) by using conventional sputter deposition (KYKY Technology Development Ltd.). The thickness of the gold film was 30 nm (0.1 nm/s, Ar gas), and the sputtering pressure was 1.0 Pa. We also deposited a thin layer of SiO_2_ (20 nm) on the gold film to reduce the chance of electrical current leakage through the sample solution. We then placed the gold-coated cover glass on a Nikon inverted microscope (TE2000-U) for device fabrication. A femtosecond (fs) laser (peak power 170 kW at 800 nm, Tsunami, Spectra-Physics) was coupled to an objective (40×, numerical aperture 0.6), with which the fs laser was focused at the SiO_2_-coated gold film with a spot diameter of 5 μm. Localised ablation of the thin film followed and the setup was effectively operating as a laser direct-write scheme. Through the use of a two-dimensional stage (OASIS controller, Objective Imaging Ltd.), we were able to selectively define electrically conductive patterns in the gold film (see images shown in Fig. 1). The final METH device structure, which typically contains a narrow current constriction, readily produces localised electrical heating akin to that due to absorption of a focused laser spot^14^,^15^,^16^. Figure 1(b) shows two parallel insulating lines of exposed glass with a width of 2.1-μm, and the endpoints separated by 5 μm. This structure corresponds to a patch METH (length 2.1 μm and width 5 μm). In addition, in order to study the variations of thermal distribution along the device, we also fabricated long rectangular heating structures with lengths 50 μm and 100 μm. Optical microscopy images of a 50 μm × 5 μm METH device is shown in Fig. 1(c).

### Colloidal particles trapping

**Figure 2:**
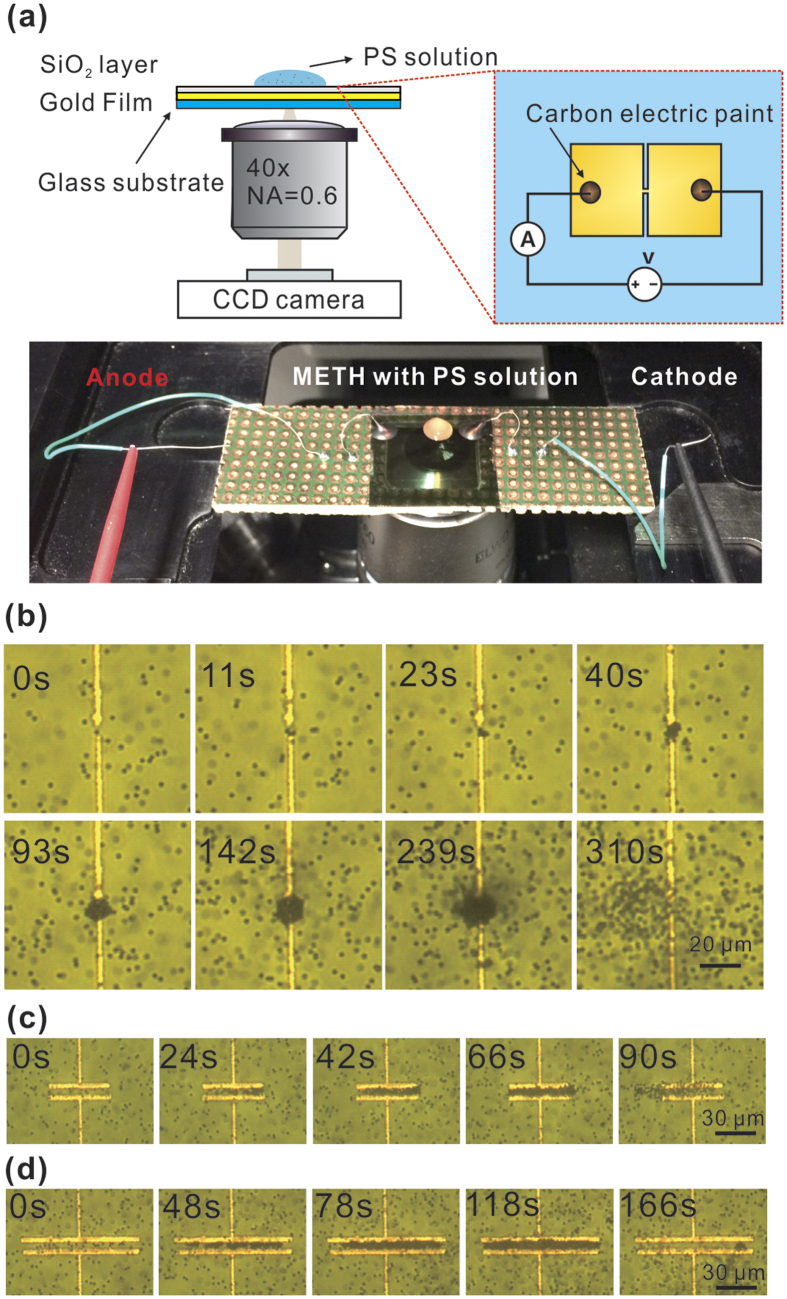
(**a**) Setup of thermal tweezers based on METH. Successive trapping image frames of 1.5 μm PSs on: (**b**) point-like METH at 30 mA; (**c**) 50 μm long METH at 20 mA; (**d**) 100 μm long METH at 16.8 mA. The power source was switched off immediately after capturing the second last image.

**Figure 3:**
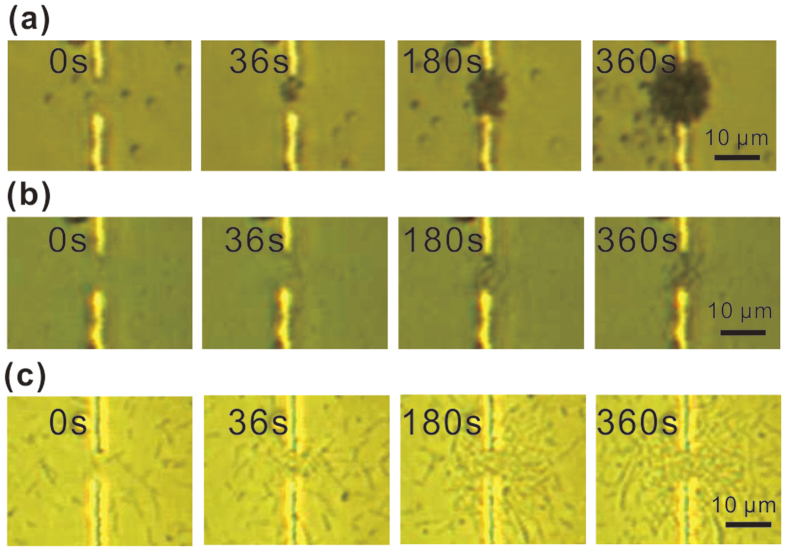
Successive trapping image frames. (**a**) Trapping of 1.0 μm PSs at 30 mA. (**b**) Trapping of 0.5 μm PSs at 30 mA. (**c**) Trapping of E. coli cells at 24 mA. (The images were captured with a CCD camera fitted with a 40× objective).

Figure 2(a) shows the overall construction of our experimental platform, with a 20 μL droplet of solution containing 1.5-μm polystyrene spheres (Polysciences Inc.) covering the METH device. As we gradually increase the supply voltage to 0.2 V, which corresponds to a current of 30 mA, localized heating is observed immediately. A convective vortex, which continuously carries the PSs from a distance to the hot region directly above the METH device, becomes clearly observable. When the PSs arrive at the hot region, as shown in Fig. 2(b), they no longer follow the convective flow and the PSs start to pile-up. After 5 minutes, the assembly reaches a steady size of ~20 μm in diameter. We then switch off the power source, which results in the release of the PS assembly through Brownian motion. As shown in Fig. 2(c,d), trapping with long METH devices is also achieved, with the maximum number of trapped particles proportional to the patch area of the device. In addition, as shown in Fig. 3(a,b), the METH device is capable of trapping of 0.5 μm and 1 μm particles. However, our current thermal tweezers are not able to trap larger particles (3–10 μm). While further experiments are on-going we attribute these results to the fact that thermophoresis is susceptible to particle size effects^35^. For larger particles, at current trapping temperature, the thermophoretic force exerted on them is not strong enough to overcome the axial drag force due to convective flow, so stable trapping is not possible.

### Bacterial cells trapping

Recently, manipulation of both single and massive *E. coli* using a tapered fiber probe have been reported by Xin *et al.*^40^,^41^. Due to the simplicity and high precision of this method, they also realized the formation of biophotonic waveguides with *E. coli*^42^. Here, we have also investigated the prospect of using this thermal-induced tweezers for trapping living biological targets. *E. coli*, the commonly used gram-negative bacteria, has been trapped with our METH structure. *E. coli* was cultured in Lysogeny broth (LB) medium and grown at 37 °C at 200 rpm. Cell passage at late exponential growth phase was performed to maintain the viability of the cell for long-term study. As shown in Fig. 3(c), under the current level of 30 mA, trapping of single-cell *E. coli* occurs within 36 s, while trapping of chain-like *E. coli* occurs at both 180 s and 360 s. The chain-like *E. coli*, with size much larger than single cell of ~1 μm, is formed due to interaction between two cells. It is expected the prolonged trapping period increase the strength to attract larger size chain-like *E. coli*. Since E. coli has a rod shape in structure, the width of chain-like E.coli is similar to single E.coli of ~0.5 μm, which is below the upper size limit current thermal tweezers can work.

To assess the possibility of trapping-induced cell damage, we measured reactive oxygen species (ROS) in the system. Oxidative stress has direct relevance to damages in DNA and protein, and therefore can be used as a tool to reveal the level of cell damage. In our experiments, the cells were pre-stained with 10 μM H_2_DCFDA (Life Technologies) according to manufacturer’s protocol before trapping. Fluorescence of 530 nm was measured after trapping, which was used to assess ROS generation. Measurement of membrane integrity loss is a method to evaluate cell survival. The bacteria after trapping were stained with both DNA binding dyes membrane freely permeable 5 μM SYTO-9 (green fluorescence) and leaky membrane permeable 30 μM propidium iodide (PI) (red fluorescence). Calculation of green/red ratio (530/630 nm) reveals the degree of membrane integrity. To study the overall cell survival and growth inhibition, bacterial cells with or without trapping were incubated in fresh LB medium and OD 600 representing cell density was measured at different time interval. To evaluate the long-term functional properties after trapping, we studied cellular methionine transfer RNA (tRNA^met^) expression level using reversed transcription-quantitative PCR (RT-qPCR). This result is a direct indicator of the ability on protein translation machinery, as its initiation requires tRNA^met^ to bind to messenger RNA that starts codon AUG. Superscript III reverse transcriptase (Life Technologies) was used to reverse-transcript the tRNA^met^ into cDNA from total RNA extracted by TRIZOL (Life Technologies), and SYBR Green Real-Time PCR Master Mixes (Life Technologies) was used to perform real-time qPCR according to manufacturer’s protocol with the primers (forward: 5′-CGCGGGGNGGAGCAGC-3′; reverse: 5′-TGGTTGCGGGGGCCG-3′). Measurement of green fluorescence signal from genetically engineered *E. coli* with green fluorescence protein (GFP) using pEGFP vector was performed to assess possible transcriptional and translational change after trapping. Change in GFP gene transcription into mRNA and mRNA translation to protein to alter GFP amount. Also, loss of fluorescence due to gene mutation or protein denaturation will decrease the green fluorescence signal.

## Results and Discussion

**Figure 4:**
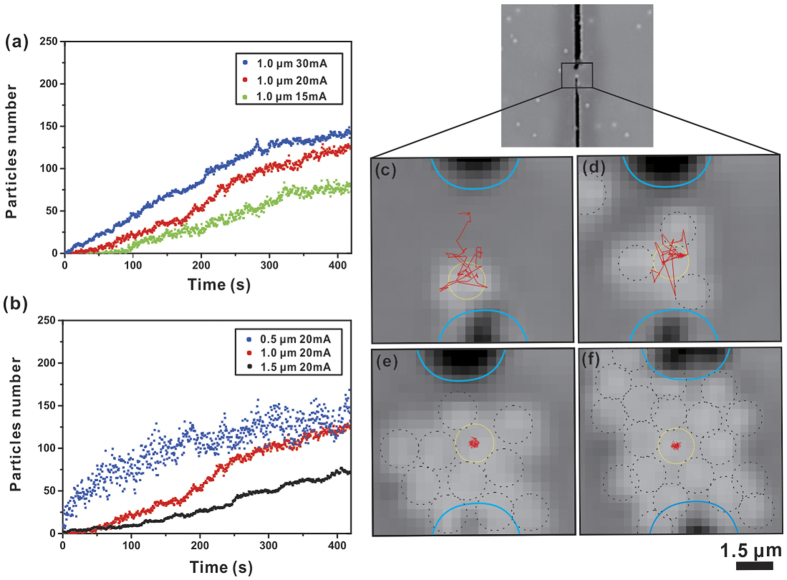
(**a**) Experimental time evolution of trapped particles number in different current levels. (**b**) Experimental time evolution of trapped particles of various sizes in same current level. Single 1.5 μm particle trajectories (shown in red line) within the METH region: (**c–f**) 1, 4, 11 and 20 particles are trapped at the time stamp of 33 s, 60 s, 119 s and 141 s, respectively. Time interval for the trajectory in each frame is 21 seconds. Black dashed circles indicate the trapped particles, and yellow dashed circle indicates the initial location of the trapped particle.

We have developed a particle-counting program based on Matlab to analyse video clips obtained from the trapping experiments frame by frame. The counting algorithm first obtained the pixel count of the trapped particle cluster in each frame of the first 500 frames (within 150 s) as a single layer of particle cluster has reasonably good image resolution. Then we obtained a linear relationship between the pixel counts and the number of particles. Using this relationship we calculated the particle numbers of other frames. Furthermore, we also observed more than one layer of particles centrally aggregated along the axial direction. The number of layers obviously depends critically on the size of the particle. Since the system is in dynamic equilibrium between convection and thermophoresis, particles at the edge of the trap may have a finite probability to move in and out of the cluster. The top-most particles are constantly vibrating because of Brownian motion and they may even undergo particle exchange with surrounding ones, thus making it not reliable to obtain an accurate particle count. 3D particle counting therefore was not conducted. We only completed 2D particle tracking of the bottom layer by analysing the pixel count of the trapped particle cluster in each frame. Particles in the upper layers are not included. We believe that this procedure should be adequate for the purpose of comparing relative trapping performances at different current levels. As revealed from Fig. 4(a), the number of trapped particles reaches an equilibrium state in approximately 7 minutes and a higher current level will result in a higher trapping rate. The time evolution of particle count in METH trapping is different from that due to optically induced thermal trapping. In the optical case, the trapping potential well takes a Gaussian shape because of the Gaussian intensity profile of the laser focal spot. Consequently, the time evolution plot of particle count increases exponentially^14^,^16^. While the METH trapping potential well is likely to take a square shape, i.e. non-Gaussian, as the temperature distribution is directly related to the shape of the current-carrying strip. Our experimental results reveal that the number of trapped particles increases linearly before settling to a steady state (see Fig. 4[a]). In addition, trapping of particles with different sizes has been investigated. Our results show that for the same current level smaller particles have higher trapping rate and larger quantity (see Fig. 4[b]). Data on the number of trapped 0.5 μm PSs are notably more scattered than the data of the other two sizes as 0.5 μm PSs are more susceptible to external disturbances such as Brownian motion. The thermal motion of the object increases with decreasing object size owing to a reduction in the viscous drag, thereby, makes it easier for them to escape. In addition, we also demonstrate trapping of single particle. We recorded the time-trajectory of a trapped particle over a time interval of 21 seconds (the duration is only limited by the video capture and image analysis software) using SpotTracker algorithm together with ImageJ software^43^. Our experimental results are shown in Fig. 4(c–f). As seen from Fig. 4(c), the trajectory of a single particle trapped by METH is typically within micrometer scale. It must be mentioned that the size of the trap is primarily related to the size of the heater element. Moreover, we also tracked the trajectory of a single particle confined by a cluster of several particles as the trap volume can readily accommodate a number of particles. As shown in Fig. 4(d–f), the movement of this particle, which has been confined to a much smaller volume as compared to the case of trapping a single particle only. We attribute the increase in particle localisation to the presence of a long range attraction between charged particles. Inter-particle forces have been studied in by Leonardo *et al.* and it was found that such forces may enhance stability of the trap^44^.

**Figure 5:**
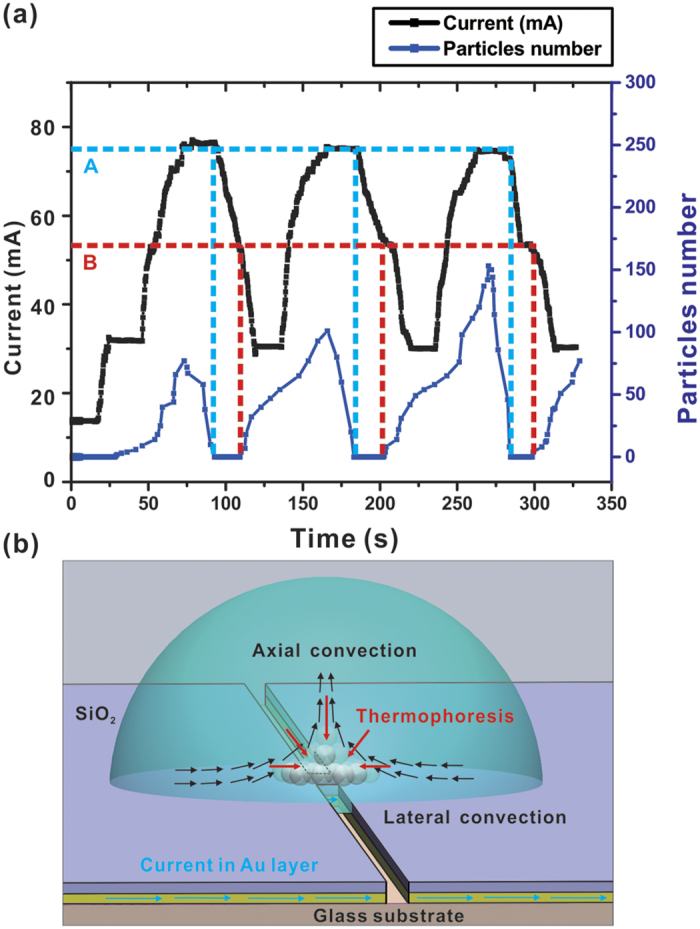
(**a**) Number of trapped particles and applied current versus time for several periods in the “trap-push-trap” experiment. Point A: critical pushing current level; Point B: critical trapping current level. Current level was recorded by a source meter (Keithley 2612B). (**b**) Force components in the trapping scheme. Thermophoresis, convective force and current are represented by arrows in red, black and blue, respectively.

In the trapping experiment, as shown in Fig. 5, current flowing through the METH device is initially 30 mA, and an increase in current induces a strong convective flow. When the current is increased to a critical level (critical pushing point A), thermophoretic force is not sufficient to overcome the axial convective drag force, and the particles are pushed away from the gold structure by a strong convective flow. Further increase the current to approximately 100 mA results in the formation of a bubble on the strip. At a steady current level just above the critical level point A, trapped particles will be continuously pushed away from the device by the notably high level of convective flow. Reducing the steady current back to a critical trapping Point B which below the critical point A will lead to the formation of a trap again and particles start to assemble above the strip. At this point, thermophoretic force is slightly greater than the axial convective drag force. Figure 5(a) shows this so-called “trap-push-trap” experiment, which has been repeated several times within the experiment, and the particle count goes through oscillatory cycles accordingly. And a schematic diagram of these force components in the trapping experiments is presented in Fig. 5(b). By comparing the two plots shown in Fig. 5(a), the following conclusions can be drawn: (i) trapping starts from a DC current of 22 mA, and convective flow is not observable at this current level, thus making it difficult to calculate the convective dragging force at this current level; (ii) increasing the supply current also means increasing temperature gradient, which in turn enhances trapping rate; (iii) as the important finding in the present context, the experimental plots of convective force and thermophoretic force as a function of temperature highlight that there exists a critical current level at which thermophoresis and axial convective drag forces are in balance.

To explain our analysis, we first look at the steady-state heat equation that describes the heat distribution around the METH device:




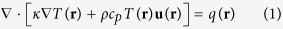




Also, the motion of fluid is governed by the incompressible Navier-Stokes equation^45^:




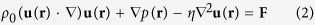




where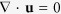.

**Figure 6:**
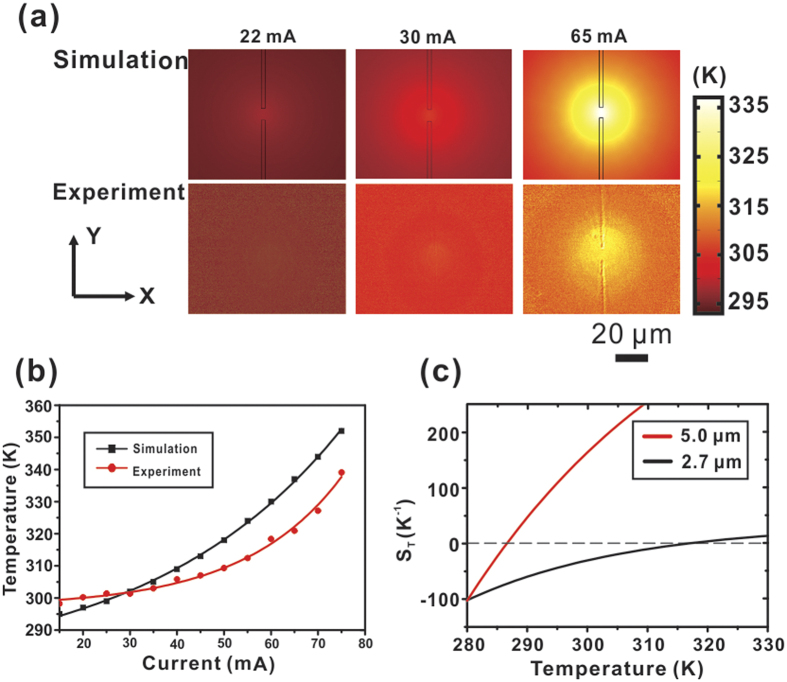
Simulation and experiment results. (**a**) Temperature distribution around the point-like METH under the critical trapping current level of 22 mA (minimum trapping current level), 30 mA (typical trapping current level), and 65 mA (critical trapping current level). (**b**) Temperature variation at different input current levels. The two lines are the exponential fit of the two data respectively. (**c**) Temperature dependence of Soret coefficient of 5.0-μm and 2.7-μm diameter particle obtained from ref. 28.

Here T(**r**), **u**(**r**) and p(**r**) refer to spatial temperature, fluid velocity and pressure distributions, respectively. And the material coefficient term κ, ρ, c_p_, and η are thermal conductivity, density, heat capacity and kinematic viscosity, respectively. With Joule heat per unit volume *q*(**r**) in Equation (1) readily solved in the METH circuit, these two equations are sufficient to describe the heat induced trapping phenomenon. The term **F** represents the force per unit volume exerting on the fluid element. The widely used Boussinesq approximation, which accounts for temperature dependence of the fluid density, is applied here^12^,^29^,^30^ by adding a buoyancy-driven convection force in the vertical direction (positive z-direction):




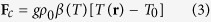




where g and ρ_0_ are gravitational acceleration and density of water, β(T) is the temperature-dependent thermal expansion coefficient of water, which is an increasing function of T. On the other hand, in terms of thermophoresis, the ∇T along z direction leads to a steady-state particles concentration gradient given by 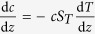, which means negative Soret coefficient S_T_ drives the particles to the hot region while positive S_T_ pushes the particles to the cold region^31^. In addition, based on extensive experimental results obtained from different systems, Piazza and his co-workers have introduced a common phenomenological fitting expression to describe the temperature dependence of the Soret coefficient^36^




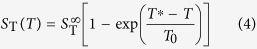




where 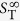, T^*^ and *T*_0_ are fitting parameters that can be calculated from experimental data. *T* is the particle ambient temperature. It is worth noting that T^*^ represents the temperature where S_T_ switches sign, which means at this temperature, the colloid particles switch from thermophilic to thermophobic. To make it more intuitive, Fig. 6(c) exhibits two typical fitting curves of *S*_T_(*T*) with the diameter of 5-μm polystyrene particles and 2.70-μm melamine particles^38^, which shows that Soret coefficient increases with increasing temperature. And the respective T^*^ is 286.6 K and 316.7 K. In addition, as the Soret coefficient has linear dependence of particle size^35^, smaller colloidal particles have larger T^*^. Therefore, one can make an estimation that the T^*^ for 1.5-μm PSs in our system is higher than 316.7 K. This theoretical prediction is in good agreement with our experimental data presented in Fig. 6(a), which shows the temperature distribution at the critical pushing level, i.e. the maximum temperature in the solution above the patch type METH device is 338 K. This temperature is closer to T^*^ of 1.5-μm PSs. Therefore, as temperature increases, the negative S_T_ gradually approaching to zero, the inward thermophoretic force that counteracts with the outward convective force gradually diminishes. Simultaneously, as Equation (3) indicates, the outward convective pushing force becomes larger. So in our experiments, after increasing the temperature beyond a certain level, the particles are pushed away by the net force of thermophoresis and axial convective forces, i.e. particles are no longer trapped.

An averaged critical trapping current I_c_ of 65 mA is experimentally derived from repeating the experiment for 50 periods. Furthermore, by analysing the trapping video frame by frame, we obtain an average axial convective velocity above the centre of the strip by making the assumption that, because of fluid continuity, the lateral arriving velocity in the vicinity of the strip is the same as the vertical convective flow directly above the region^28^,^29^. Therefore, we first calculate the axial convective drag force F using the Stokes law: F = μv, where μ = 3πεηD is the drag coefficient with a correction factor ε = 2.67 by considering the particle proximity to the substrate^46^, η is the dynamic viscosity and D is PS diameter. Experimental data obtained from 50 trapping experiments suggest that the averaged particle velocity at critical pushing level v_t_ is 5.20 μm/s, and the averaged particle velocity at critical trapping level v_p_ is 3.30 μm/s. Here, we take v = (v_t_ + v_p_)/2 = 4.25 μm/s to be the critical point of this METH device. This calculation approach is more accurate because it has taken into consideration the discrepancy of the data at critical pushing and critical trapping points. And at this critical trapping current I_c_, the highest temperature in the METH is 338 K (see Fig. 6), and at this temperature, the dynamic viscosity η = 0.43 × 10^−3^ Ns∕m^2^ according to ref. 47. Therefore, the critical convective drag force for trapping 1.5-μm PS is 68.95 fN, which is within the same magnitude order of thermophoretic force. Our result is in agreement with the thermophoretic force reported in ref. 38, which ranges from 20 to 100 fN.

**Figure 7:**
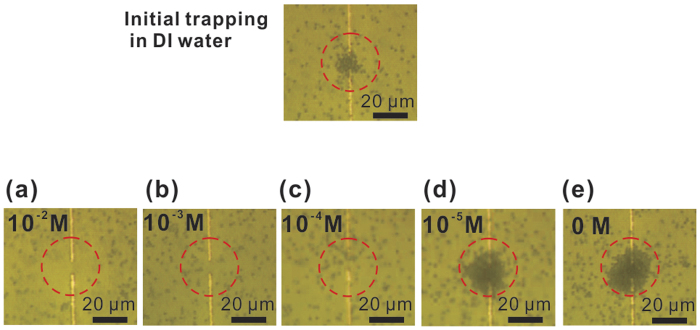
(**a**–**e**) The trapped 1.5 μm particles diffused into the solution after adding NaCl solution with various concentration, the trapping current is 30 mA, the diameter of the dashed circle is 35 μm.

Furthermore, it is reported that thermophoretic force changes with ion concentration^37^,^48^, while the buoyancy driven Rayleigh-bénard convection is not so susceptible to such changes^14^,^15^,^16^,^28^. To verify this, after establishing steady trapping of a cluster of PSs, we injected a small drop (2 μL) of NaCl solution into the 20 μL PS solution. And a series of samples with different NaCl concentration levels have been tested. When the ions diffused into the trapped particles cluster, the PSs were released almost immediately although the current remained the same. The particles are not trapped again no matter how we change the supply current, and we could only observe the inward and outward convective flow (see Fig. 7). Below a critical concentration value (10^−5^ M for 1.5 μm PSs), particle trapping still exists, but higher than this level, particles are released. In addition, as shown in Fig. 7, the PSs concentration in red dashed line circle (r = 12 μm) decreases with increasing NaCl concentration. Particularly as shown in Fig. 7(a), a high NaCl concentration (10^−2^ M) leads to a void region where most particles are radially pushed away from the hot area. The PSs we used contain a slight anionic charge from sulfate ester. Consequently, as NaCl concentration increases, the Seebeck effect in the electrolyte, may lead a change in the S_T_ of negatively charged particles from negative to positive^48^,^49^,^50^, thus alters the associated thermophoresis from thermophilic to thermophobic. So under such high NaCl concentration levels, the particles originally trapped in the hot centre region are driven to the surrounding cold region through positive thermophoresis. In contrast, the bacteria trapping experiments were performed in LB medium, where approximately 10^−1^ M NaCl was present. The reason of successful trapping of live E. coli in the LB medium could be due to the cell’s tendency to maintain negative membrane surface charge so as to restore its thermophilic property. On the other hand, successful trapping of heat-killed E. coli in LB medium might be explained by the presence of water-soluble proteins from yeast extracts and other charged components in the LB medium, which can neutralize the change in bacterial surface charge. Nonetheless, further investigation is required on why living organism can be trapped under high levels of NaCl concentration. Nonetheless, our experimental results indicate that thermophoretic force is greatly influenced by ion concentration, and thermophoresis plays a key role in the reported thermal trapping scheme.

Within our experiments, we have measured the temperature distribution of the patch type METH operating at different current levels by using a fluorescence emission efficiency method. We previously obtained an experimental temperature-efficiency plot of 0.1 mM Rhodamine B solution^14^. However, this approach only offers average temperature measurements along the z-axis as the fluorescence signal is collected from the bulk solution immediately above the METH structure. Given that at steady state the system may be regarded as a 3-dimensional heat flow problem with well-defined boundary conditions, one would expect that simulative analysis may provide temperature data points, hence temperature gradients. Our METH structure was analysed using the AC/DC Module and Heat Transfer Module available from a finite-element solver COMSOL Multiphysics. As shown in Fig. 6(a), the simulation result of temperature distribution around the METH device is directly calculated from its surface. This result is higher than that obtained from experiment because the latter only provides averaged values that also cover colder regions vertically away from the heat source. The maximum temperature within the device region increases exponentially with increasing input current. Indeed, as shown in Fig. 6(b), our experimental results are in good agreement with those obtained from numerical simulations.

**Figure 8:**
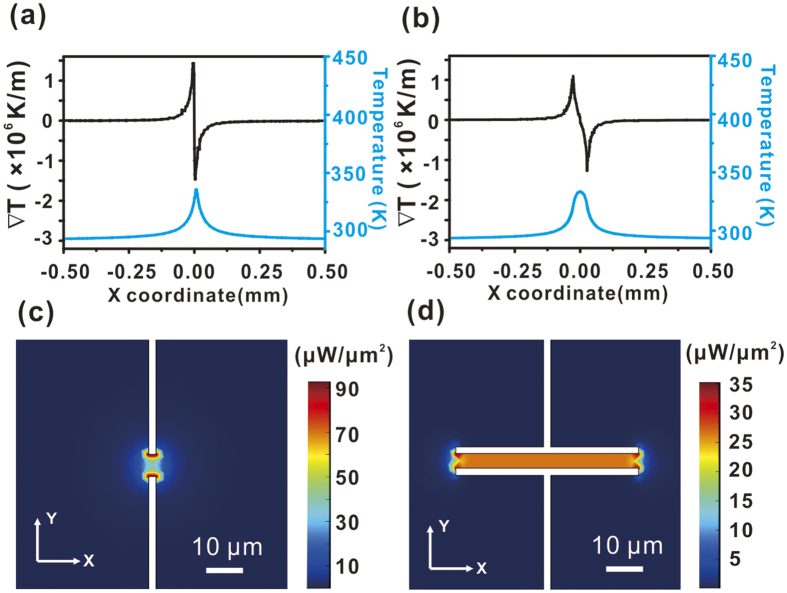
Temperature distribution (blue line) and temperature gradient (black line) at line y = 0 (coordinate origin is at the center of the METH structure). (**a**) Point-like METH driven at a current level of 65 mA. Temperature gradient peak-to-peak distance = 10 μm, temperature FWHM = 50.1 μm. (**b**) 50 μm long METH driven at a current level of 30 mA. Temperature gradient peak-to-peak distance = 55 μm, temperature FWHM = 71.7 μm. (**c**) Power density on point-like METH at 65 mA. (**d**) Power density in 50-μm long METH driven at 30 mA.

The temperature gradient distribution along the X coordinate was also calculated. As shown from Fig. 8(a,b), the temperature gradient reaches its maximum at the vicinity of device’s narrowing edge, the distance between two temperature gradient peaks of the patch type METH (10 μm) is smaller than the long rectangular type (50-μm long) METH (55 μm). The temperature distribution is in size and shape dependence of METH. And the sign of _∇*T*_ changes from positive to negative at x = 0. In our experiment, the sign of S_T_ is negative, hence leading to the consequence of particles are trapped within the hot region. In addition, as shown by the temperature profiles in Fig. 8, the FWHM of the patch type METH is 50.1 μm, while at same power intensity level, the optically induced thermal tweezers provide a narrower temperature distribution (10 μm–20 μm), consequently resulting in a much higher temperature gradient^14^,^15^,^16^,^22^. This also explains the current observation that METH induced tweezers have lower trapping force as well as trapping speed as compared to their optical counterparts. Here, we have also calculated the electric power density. As shown in Fig. 8(c,d), a typical threshold power density for trapping ranges from 10 μW/μm^2^ to 100 μW/μm^2^.

**Figure 9:**
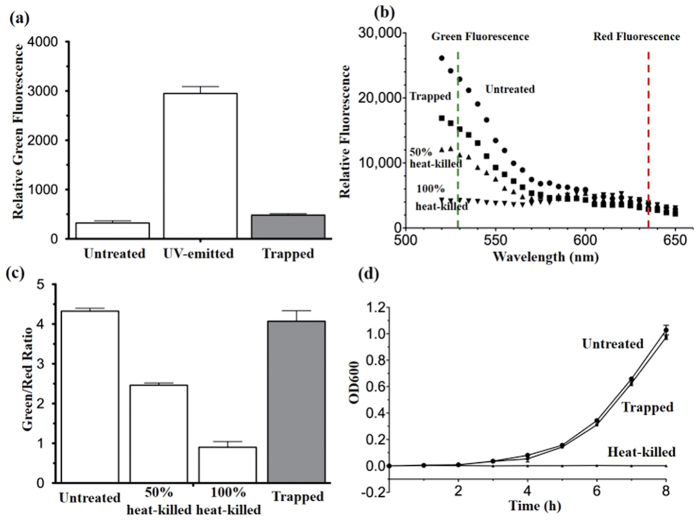
Effect of trapping on bacterial viability. (**a**) Fluorescence signal on ROS generation with or without trapping treatment and UV-emitted bacteria, which is the positive control set by 8 W ultraviolet lamp (254 nm) emission on bacterial cell for 1 min to increase ROS level dramatically. (**b**) Fluorescence emission spectrum of the stained E. coli with or without trapping treatment, with heat-killed bacteria control. (**c**) Green/Red fluorescence ratio (530 nm/635 nm) from (**b**). (**d**) Bacteria growth rate. Results are mean ± SEM (n = 3 independent experiments).

**Figure 10:**
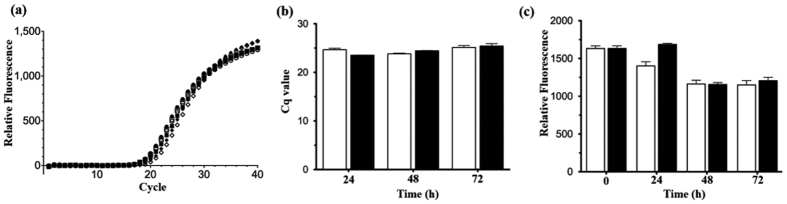
Effect of trapping on long-term bacterial function. (**a**) Fluorescence plots of real-time PCR on tRNA^met^ of untreated (white) and trapped (black) bacteria at 24 h (circle), 48 h (square) and 72 h (triangle). (**b**) Analysis of tRNA^met^ relative expression level in term of Cq (cycle of fluorescence over threshold 500 units) between untreated (white) and trapped (black) bacteria after experiment from 24 to 72 h. (**c**) Fluorescence signal from green fluorescence protein between untreated (white) and trapped (black) bacteria after experiment from 0 to 72 h. Results are mean ± SEM (n = 3 independent experiments).

To trigger a trap, the minimum power density at the centre of patch type METH is 10.6 μW/ μm^2^ (22 mA) and 5.6 μW/ μm^2^ (12 mA) for the 50-μm long METH, which is quite low and it is confirmed that such a low heating power did not induce any harmful effects to cell viability as long as the current level is properly controlled (under 30 mA). For real-time ROS measurement, 9.6 ± 0.9% ROS generation is observed in bacteria cells by comparing the fluorescence between negative and UV-emitted bacteria control shown in Fig. 9(a). Also, there are 6.0 ± 4.5% bacterial cells with loss of membrane integrity after trapping shown in Fig. 9(b,c). Moreover, the growth rates between untreated control and bacteria after trapping are similar, and there is as no growth in heat-killed bacteria, suggesting no growth inhibition and significant cell death occur after trapping. These results indicate that the bacteria intracellular replication mechanism was not disrupted by the trapping process. For long-term study of the bacteria after trapping, the relative expression level of tRNA^met^ is similar between untreated and trapped bacteria up to 72 h, suggesting that the mRNA translational machinery is intact after trapping (Fig. 10(a,b)). Also, green fluorescence signal remains similar as the fluorescence value is insignificant difference between untreated and trapped bacteria up to 72 h, suggesting that the synthesis rate of green fluorescence protein remains unchanged and no obvious change of protein characteristics after trapping (Fig. 10(c)). Therefore, one can readily use METH to trap the living cells for downstream applications without causing cell damage or functional change.

In summary, our METH device fabricated from a continuous gold thin film has shown good trapping capability. With the possibility of massive scaling into 2-dimensional arrays, the electro-thermal approach offers a useful alternative to conventional optical tweezers. In our experiments, the devices were conveniently fabricated by direct writing with a femtosecond laser. Trapping of dielectric particles (0.5–1.5 μm) as well as live *E. coli* have been demonstrated. The METH approach is based on the combined effect of thermal convection and thermophoresis. The threshold power density for generating a trap is well below 100 μW/ μm^2^, which is lower than the power level commonly achievable in plasmonic optical tweezers^6^,^9^,^11^. The thermophoretic force exerted on the 1.5 μm PS at the critical trapping point has been calculated with the help of simulation and experimental data from which we obtain the temperature distribution and temperature gradient around the METH device. Furthermore, it is well known that optical tweezers are inefficient in their ability to manipulate particles which have a refractive index comparable to their surrounding medium, or particles that are non-transparent and optical sensitive. Moreover, isolated plasmonic nanostructures are usually not capable of producing fast mass transportation at high fluid velocity (>10 nm/s) in micro- or nano-fluidic environment^30^. The reported thermal induced tweezers can overcome these limitations while at the same time free from cumbersome optical setup. Moreover, their lower threshold power density renders lower risk of causing harmful effects on biological samples. Its planar construction also makes the device very suitable for integration with microfluidics and lab-on-chip systems.

## Additional Information

**How to cite this article**: Chen, J. *et al.* Thermal gradient induced tweezers for the manipulation of particles and cells. *Sci. Rep.* **6**, 35814; doi: 10.1038/srep35814 (2016).

**Publisher’s note**: Springer Nature remains neutral with regard to jurisdictional claims in published maps and institutional affiliations.
